# *In-vitro* and *in-silico* antibacterial activity of *Azadirachta indica* (Neem), methanolic extract, and identification of Beta.d-Mannofuranoside as a promising antibacterial agent

**DOI:** 10.1186/s12870-022-03650-5

**Published:** 2022-05-25

**Authors:** Hisham N. Altayb, Nijood F. Yassin, Salman Hosawi, Imran Kazmi

**Affiliations:** 1 Biochemistry Department, Faculty of Science, King Abdulaziz University, Jeddah 21589, Saudi Arabia; 2 Centre for Artificial Intelligence in Precision Medicine, King Abdulaziz University, Jeddah, 21589, Saudi Arabia; 3 Department Microbiology, College of Medical Laboratory Sciences, Sudan University of Science and Technology, Khartoum, Sudan

**Keywords:** Antimicrobial resistance, *.*beta.d-Mannofuranoside, O-geranyl, GC–MS, Molecular docking, Bioactive compounds, Sudan

## Abstract

**Background:**

Antimicrobial resistance became the leading cause of death globally, resulting in an urgent need for the discovery of new, safe, and efficient antibacterial agents. Compounds derived from plants can provide an essential source of new types of antibiotics. *A. indica* (neem) plant is rich in antimicrobial phytoconstituents. Here, we used the sensitive and reliable gas chromatography-mass spectrometry (GC–MS) approach, for the quantitative and quantitative determination of bioactive constituents in methanolic extract of neem leaves grown in Sudan. Subsequently, antibacterial activity, pharmacokinetic and toxicological properties were utilized using in silico tools.

**Results:**

The methanolic extract of neem leaves was found to have antibacterial activity against all pathogenic and reference strains. The lowest concentration reported with bacterial activity was 3.125%, which showed zones of inhibition of more than 10 mm on *P. aeruginosa, K. pneumoniae, Citrobacter* spp., and *E. coli,* and 8 mm on *Proteus* spp., *E. faecalis, S. epidermidis*, and the pathogenic *S. aureus.* GC–MS analysis revealed the presence of 30 chemical compounds, including fatty acids (11), hydrocarbons (9), pyridine derivatives (2), aldehydes (2), phenol group (1), aromatic substances (1), coumarins (1), and monoterpenes (1). In silico and in vitro tools revealed that.beta.d-Mannofuranoside, O-geranyl was the most active compound on different bacterial proteins. It showed the best docking energy (-8 kcal/mol) and best stability with different bacterial essential proteins during molecular dynamic (MD) simulation. It also had a good minimum inhibitory concentration (MIC) (32 μg/ml and 64 μg/ml) against *S. aureus* (ATCC 25,923) and *E. coli* (ATCC 25,922) respectively.

**Conclusion:**

The methanolic extract of *A. indica* leaves possessed strong antibacterial activity against different types of bacteria. Beta.d-Mannofuranoside, O-geranyl was the most active compound and it passed 5 rules of drug-likeness properties. It could therefore be further processed for animal testing and clinical trials for its possible use as an antibacterial agent with commercial values.

**Supplementary Information:**

The online version contains supplementary material available at 10.1186/s12870-022-03650-5.

## Background

Medicinal plants are known to have a wide range of bioactive compounds that have antimicrobial, antifungal, anticancer, anti-inflammatory, and antioxidant activities [1–3]. Many researchers have documented the potent activity of plants’ bioactive compounds on drug-sensitive and resistant bacteria [4–6]. Although plants contain a very large number of bioactive compounds, few have been discovered [1]. The development of extraction methods and the use of molecular spectroscopic techniques such as GC–MS and fourier-transform infrared (FTIR) has led to the discovery and characterization of new plant bioactive compounds [1, 7–9]. Recently, in silico tools have emerged as promising, time and cost-saving approaches for drug discovery [10].

*Azadirachta indica* (*A. indica*) is one of the Meliaceae family known as neem. It has been used in traditional medicine since ancient times to treat a range of human diseases [11]. The leaves, seeds, and roots of neem contain antibacterial and antifungal agents [12, 13]. This biological activity of neem stems from many bioactive compounds that are structurally and chemically diverse, with more than 140 compounds found in different parts of the plant [14]. Several types of biological compounds are extracted from neem, including ketones, carotenoids, flavonoids, steroids, and phenolic compounds [15]. The antibacterial activity of *A. indica* leaves extract has been documented on different bacterial species including *E. coli*, *Staphylococcus* species, *Streptococcus* species, and *Pseudomonas* species [16–18].

Antimicrobial resistance is one of the major problems facing global health today [19], and it is a major source of morbidity and mortality globally [20]. Nowadays it is becoming the leading cause of death globally [21]. A large number of bacteria have acquired and developed antimicrobial resistance mechanisms [20], which constitutes a burden on the global health system with the increasing financial cost [22]. With the increase in drug resistance, there are very few alternatives for patients, and as a result, the number of deaths associated with it has increased [23]. In America, there are 23,000 deaths annually related to drug resistance [20]. The emergence of infectious diseases and the development of antibiotic resistance in bacteria resulting in decreased action or failure of existing antibacterial agents [24], has resulted in an urgent need for the discovery of new, safe, and efficient antibacterial agents [25]. Compounds derived from plants can provide an essential source of new types of antibiotics. There are many types of phytochemicals of plant extract that can exert potential activity on sensitive and multidrug-resistant bacteria [24, 26].

Although a number of studies have reported various neem bioactive compounds, there hasn't been much focus on.beta.d-Mannofuranoside, O-geranyl. Scanty information is available on this compound. In a study conducted in India, the authors documented the presence of this compound in mangroves associates crude extract with antimicrobial activity [27], and in another study conducted by Iga et al., they used the isomers of synthetic D-mannofuranoside as antiallergic and anti-inflammatory agents [28]. The present study focused on the extraction, GC–MS analysis, and investigation of the antimicrobial activity of crude neem methanolic extract on drug-resistant and sensitive bacteria. Subsequently, the molecular docking and MD simulation studies were explored for the evaluation of the activity of GC–MS-identified compounds on different bacterial essential proteins. Accordingly, in vitro study was explored for the analysis of the antibacterial activity of the pure.beta.d-Mannofuranoside, O-geranyl compound.

## Results

### Bacterial isolates

From 130 urine samples, 100 bacterial isolates were identified. From the 100 bacterial isolates, 90 were Gram-negative rods, and 10 were Gram-positive cocci. *Escherichia coli* represents the majority (70%) of isolates, followed by *Klebsiella pneumoniae* (9%), *Enterococcus faecalis* (8%)*, Pseudomonas aeruginosa* (5%), *Proteus* spp. (5%), *Citrobacter* spp. (1%), *Staphylococcus aureus* (1%), and *Staphylococcus epidermidis* (1%).

### Antibacterial Susceptibility tests

The antibacterial susceptibility tests showed that 81.4% of *E. coli* isolates were resistant to ciprofloxacin, 75.7% to ceftazidime, 71.4% to cotrimoxazole, 62.9% to gentamicin, and 10% to imipenem. *P. aeruginosa* showed resistance to ceftazidime (80%), ciprofloxacin (40%), gentamicin (40%), cotrimoxazole (40%), imipenem (0%). *Proteus* species showed high resistance to ceftazidime (60%), cotrimoxazole (60%) and imipenem (40%); a low resistance rate was also observed in ciprofloxacin (20%) and gentamicin (20%). *K. pneumoniae* was highly resistant to ceftazidime (66.7%), and cotrimoxazole (55.6%), the resistance rate to ciprofloxacin, gentamicin, and imipenem was 22.2%. *E. faecalis* was highly resistant to ceftazidime (50%), ciprofloxacin (37.5%), gentamicin (37.5%), imipenem (37.5%), and cotrimoxazole (25%) (Table 1).

**Table 1:** Antimicrobial susceptibility testing of commonly used antimicrobial agents on clinical isolates

Bacterial isolates	Ciprofloxacin		Gentamicin		Cotrimoxazole		Ceftazidime		Imipenem	
	S	R	S	R	S	R	S	R	S	R
*E. coli* (*n* = 70)	13 (18%)	57 (82%)	26 (37%)	44 (63%)	20 (29%)	50 (71%)	17 (24%)	53 (76%)	63 (90%)	7 (10%)
*K. pneumoniae* (*n* = 9)	7 (78%)	2 (22%)	7 (78%)	2 (22%)	4 (44%)	5 (56%)	3 (33%)	6 (67%)	7 (78%)	2 (22%)
*Citrobacter* spp. (*n* = 1)	1 (100%)	-	1 (100%)	-	-	1 (100%)	**-**	1 (100%)	1 (100%)	**-**
*E. faecalis* (*n* = 8)	5 (63%)	3 (37%)	5 (63%)	3 (37%)	6 (75%)	2 (25%)	4 (50%)	4 (50%)	5 (63%)	3 (37%)
*P. aeruginosa* (*n* = 5)	3 (60%)	2 (40%)	3 (60%)	2 (40%)	3 (60%)	2 (40%)	1 (20%)	4 (80%)	5 (100%)	**-**
*Proteus* spp*.* (*n* = 5)	4 (80%)	1 (20%)	4 (80%)	1 (20%)	2 (40%)	3 (60%)	2 (40%)	3 (60%)	3 (60%)	2 (40%)
*S. epidermidis* (*n* = 1)	**-**	1 (100%)	1 (100%)	**-**	1 (100%)	**-**	1 (100%)	**-**	1 (100%)	**-**
*S. aureus* (*n* = 1)	**-**	1 (100%)	**-**	1 (100%)	1 (100%)	**-**	1 (100%)	**-**	1 (100%)	**-**
Total (*n* = 100)	33 (35%)	67 (65%)	47 (45%)	53 (55%)	37 (37%)	63 (63%)	29 (29%)	71 (71%)	86 (84%)	14 (16%)

Abbreviation: *R*= Resistant, *S*= Sensitive, *n=* Number

### Antimicrobial activity of *A. indica*

In this study, methanolic extract of *A. indica* showed antimicrobial activity against strains of *S. aureus*, *P. aeruginosa*, *E. coli*, *Proteus* spp., *S. epidermidis*, *Citrobacter* spp., *K. pneumoniae*, *E. faecalis*, *S. aureus* ATCC 25,923, and *E. coli* ATCC 25,922. A low (6.25%) concentration of the extract showed activity on *K. pneumoniae, Citrobacter* spp.*, P. aeruginosa,* and control strains, while 12.5% concentration was active on *E. faecalis, Proteus* spp.*, S. epidermidis*. The concentration of 3.125% moderately inhibits the bacteria, with the best activity (13 ± 1.8) recorded on *P. aeruginosa.* The concentration of 1.5% was not active on all bacterial strains (Table 2) (Supplementary figures S2 and S3).

**Table 2:** Mean of inhibition zones (diameter in millimeters) after in vitro exposure of isolates to *A. indica* methanol extract in different concentrations

Methanolic extract concentrations (%)						
*Bacterial isolates*	50	25	12.5	6.25	3.125	1.5
*E. coli* (*n* = 70)	17.5 ± 1	15.5 ± 1	13.5 ± 0.5	12 ± 0.8	10 ± 0.5	N.A
*K. pneumoniae* (*n* = 9)	19 ± 1.6	17 ± 2	15.4 ± 2	13.5 ± 1.8	11 ± 1.9	N.A
*Citrobacter* spp. (*n* = 1)	22 ± 1	20 ± 1.5	18 ± 0.5	16 ± 1.1	11 ± 0.8	N.A
*E. faecalis* (*n* = 8)	16.5 ± 1.8	14.5 ± 2.2	13 ± 2.1	9 ± 1.6	8 ± 1.9	N.A
*P. aeruginosa* (*n* = 5)	21 ± 1	19 ± 0.8	17 ± 1.3	16 ± 1.5	13 ± 1.8	N.A
*Proteus* spp. (*n* = 5)	17.5 ± 1.8	15 ± 1.6	11 ± 1.2	9 ± 0.9	8 ± 0.9	N.A
*S. epidermidis* (*n *= 1)	15 ± 1.1	14 ± 1.5	13 ± 1.2	12 ± 0.5	8 ± 0.5	N.A
*S. aureus* (*n* = 1)	13 ± 0.5	11 ± 0.8	10 ± 0.7	9 ± 0.4	8 ± 0.5	N.A
*E. coli* ATCC® 25,922	18 ± 0.4	16 ± 1	14 ± 1	12.5 ± 0.4	8 ± 1	NA
*S. aureus* ATCC® 25,923	22 ± 0.8	20 ± 1.7	18 ± 2	15 ± 2	10 ± 0.5	NA

Diameters of inhibition zones were measured in millimeters. Less than 9 mm zone was considered inactive, 9–12 mm as partially active, 13–18 mm as active, and 18 mm were very active. N.A = Not active

### Gas chromatography results

GC–MS analysis of neem leaves methanolic extract revealed 30 peaks, corresponding to 30 phytochemical compounds as shown in Table 3, including fatty acids (11), hydrocarbons (9), pyridine derivatives (2) and aldehydes (2), phenol group (1), aromatic substances (1), coumarins (1), and monoterpenes (1). The 1,5-Anhydro-2-deoxy-L-arabino-hex-1-enitol was the most predominant compound with a peak area percentage of 15.6.

**Table 3:** Compounds identified in methanolic extract of neem and their docking energy

**No**	Group	Neem ingredient compounds	Area %	Compound ID	1JIJ	3TTZ	5OJ0	1TVF
1	carbohydrate	1,5-Anhydro-2-deoxy-L-arabino-hex-1-enitol	15.6	CSP: 8,686,379	-6.2	-4.8	-5.2	-5.6
2	hydrocarbons	1, 3-Propanediol, 2-(hydroxymethyl)-2-nitro-	10.34	CID_31337	-4.7	-3.9	-3.9	-5.4
3	fatty acid	3,7,11,15-Tetramethyl-2-hexadecen-1-ol	8.55	CID_5366244	-4.6	-2.9	-3.6	-3.9
4	fatty acid	n-Hexadecanoic acid	7.75	CID_985	-1.5	-3.1	-5.1	-4.1
5	monoterpenes	.beta.d-Mannofuranoside, O-geranyl	6.17	CID_5365843	-8	-8.3	-8	-9.7
6	hydrocarbons	Phytol	4.06	CID_5280435	-0.2	-2.5	-3.1	-3.9
7	hydrocarbons	4H-Pyran-4-one, 2,3-dihydro-3,5-dihydroxy-6-methyl-	3.87	CID_119838	-5.9	-3.9	-5.2	-7.6
8	hydrocarbons	Dihydroxyacetone	3.38	CID_670	-3.7	-2.7	-4.2	-4.8
9	Coumarins	Benzoofuran, 2, 3-dihydro-	3.06	CID_10329	-3.4	-3.6	3.9	-3.4
10	hydrocarbons	9-Eicosyne	2.38	CID_557019	-0.8	-1.1	-1.9	-1
11	dehydro-reducing sugars	5-Hydroxymethylfurlfural	2.03	CID_237332	-5	-4.2	-3.9	-5.1
12	hydrocarbons	1-Dodecanol	1.88	CID_8193	-0.4	-0.4	-1.9	-1.2
13	fatty acid	9,12,15-Octadecatrienoic acid, (Z,Z,Z)-	1.76	CID_5280934	-0.8	-4	-5.8	-3.9
14	fatty acids	Hexadecanoic acid*, *2*-*hydroxy*-*1*-(*hydroxymethyl)ethyl ester	1.58	CID_123409	-5.4	-5.2	-5.8	-6.8
15	fatty acid methyl esters	1, 2, 3-Propanetriol,1-acetate	1.41	CID_33510	-4.2	-3.8	3.9	-6.2
16	hydrocarbons	Phytol, acetate	1.36	CID_6428538	-1.9	-4	-4.7	-3.2
17	hydrocarbons	Furan, 2, 5-dimethyl-	1.33	CID_12266	-2.9	-3.2	-3	-2.7
18	aromatic substances	2-Methoxy-4-vinylphenol	1.29	CID_332	-4.6	-4.6	-4.4	-4.2
19	fatty acids	Dodeccanoic acid	1.23	CID_3893	-1.9	-2.1	-5.6	-1.2
20	pyridine derivatives	5-Pyrimidinol, 2-methyl-4-(methylthio)-	1.22	CID_593599	-4	-2.8	-3.6	-4
21	aldehydes	4-Methyl-2, 5-dimethoxybenzaldehyde	1.18	CID_602019	-4.8	-4.8	-5	-4.9
22	pyridine derivatives	4(H)-Pyridine, N-acetyl-	1.09	CID_556800	-4.1	-3.7	3.8	-3.7
23	phenol group	Phenol, 2, 6-dimethoxy-	1.07	CID_7041	-5	-4.7	-4.6	-4.4
24	fatty acids	Tetradecanoic acid	0.96	CID_11005	-2	-3.4	-5.1	-5.2
25	fatty acids	Octadecanoic acid	0.82	CID_5281	-0.9	-3.3	-4.4	-5.6
26	hydrocarbons	2, 5-Dimethyl-4-hydroxy-3(2H)-furanone	0.66	CID_19309	-5	-3.2	-6.5	-6.8
27	fatty acid methyl esters	Pentanedioic acid, 3, 3-dimethyl-, monomethylester	0.65	SID_249925496	-2.8	-2.9	-3.4	-4.9
28	fatty acids	Pentanoic acid, 4-oxo-	0.65	CID_11579	-3.6	-3	-4.9	-6
29	aldehydes	Octadecanal	0.64	CID_12533	0.73	-1.6	1.8	-1.8
30	fatty acid methyl esters	Hexadecanoic acid, methyl ester	0.48	CID_8181	2.37	-1	-2.5	-2

Tyrosyl-tRNA synthetase (PDB ID: 1JIJ), DNA gyrase (PDB ID: 3TTZ), Penicillin-Binding Protein 2X (PBP2X) (PDB ID: 5OJ0), and penicillin-binding protein 4 (PBP4) (PDB ID: 1TVF)

### Molecular docking

**Fig. 1.**
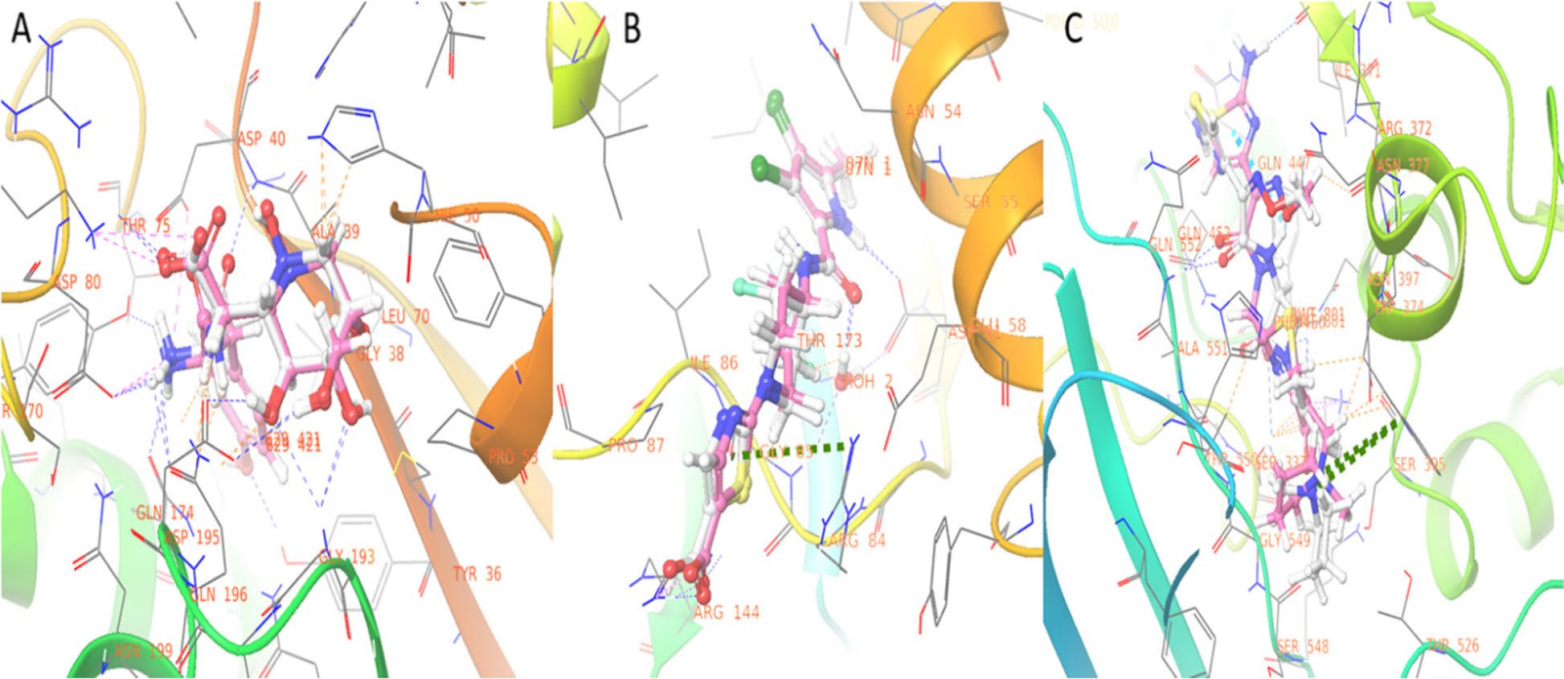
3D interaction of superimposed crystal structure of protein inhibitors and that after redocking the same inhibitors with Maestro. The dashed lines indicate dashed lines Pi-cation (**green**), H-bonds (**blue**), bad contact (**brown**), and ugly contact (**red**). Active site residues (labeled in brown) showed interaction with reference co-crystallized ligands (white) and re-docked the same ligands (violet). A. 1JIJ and SB-239629 inhibitor. B. 3TTZ and 07 N inhibitor and C. 5OJ0 and cefepime

The validation of docking protocols revealed the same orientation of redocked inhibitors with experimentally determined positions, the Root Mean Square Deviation (RMSD) between superimposed SB-239629, 07 N, and cefepime were 1.2, 0.66, and 2 Å, and docking energy -11.6, -6.8, and -8.25 kcal/mol respectively (Fig. 1).

**Fig. 2.**
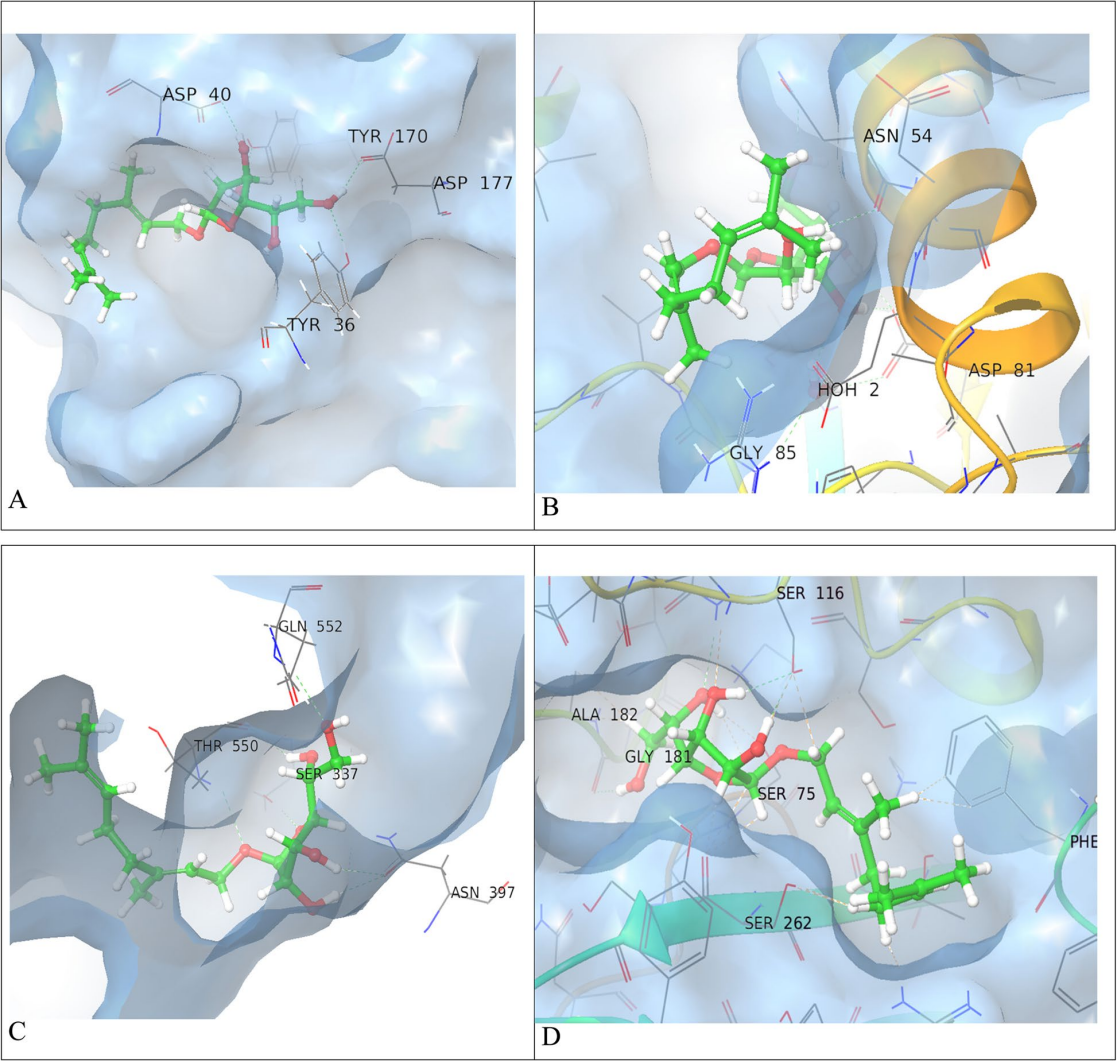
3D structures of the interaction of.beta.d-Mannofuranoside, O-geranyl (green) at protein binding sites. The protein backbones are colored blue, residues associated with the interaction are labeled in black, H-bonds (**green**), bad interactions (**brown**). A. tyrosyl-tRNA synthetase (PDB ID: 1JIJ). B. DNA gyrase (PDB ID: 3TTZ). C. Penicillin-Binding Protein 2X (PBP2X) (PDB ID: 5OJ0), and D. Penicillin-binding protein 4 (PBP4) (PDB ID: 1TVF)

**Fig. 3.**
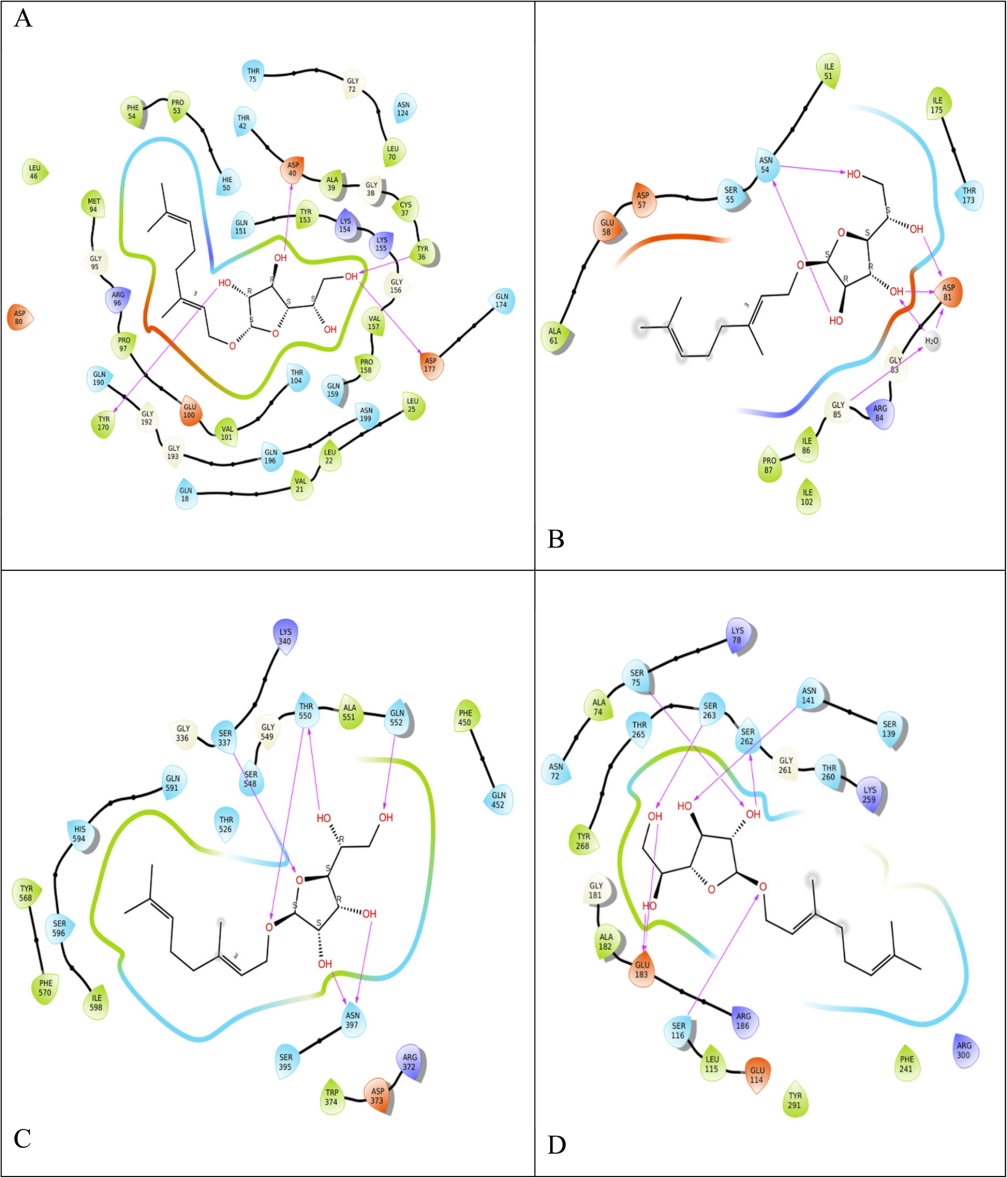
2D interaction of.beta.d-Mannofuranoside, O-geranyl (**black**) and proteins binding site residues during molecular docking. H-bonds (**violet**), pi-cation (**red**), active site residues are colored red (**negatively charged**), deep blue (**positively charged**), green (**hydrophobic**), pale yellow (**glycin**), and pale blue (**polar**) A. tyrosyl-tRNA synthetase (PDB ID: 1JIJ) B. DNA gyrase (PDB ID: 3TTZ) C. Penicillin-Binding Protein 2X (PBP2X) (PDB ID: 5OJ0), and D. Penicillin-binding protein 4 (PBP4) (PDB ID: 1TVF)

The molecular docking of GC–MS-identified compounds in neem showed variable activities on different bacterial proteins. The.beta.d-Mannofuranoside, O-geranyl showed best docking energy on the four selected bacterial proteins, -8, 8.3, -8, and -9.7 kcal/mol on tyrosyl-tRNA synthetase (PDB ID: 1JIJ), DNA gyrase (PDB ID: 3TTZ), Penicillin-Binding Protein 2X (PBP2X) (PDB ID: 5OJ0), and penicillin-binding protein 4 (PBP4) (PDB ID: 1TVF) respectively (Figs. 2 and 3).

### Molecular Dynamics simulation

**Fig. 4:**
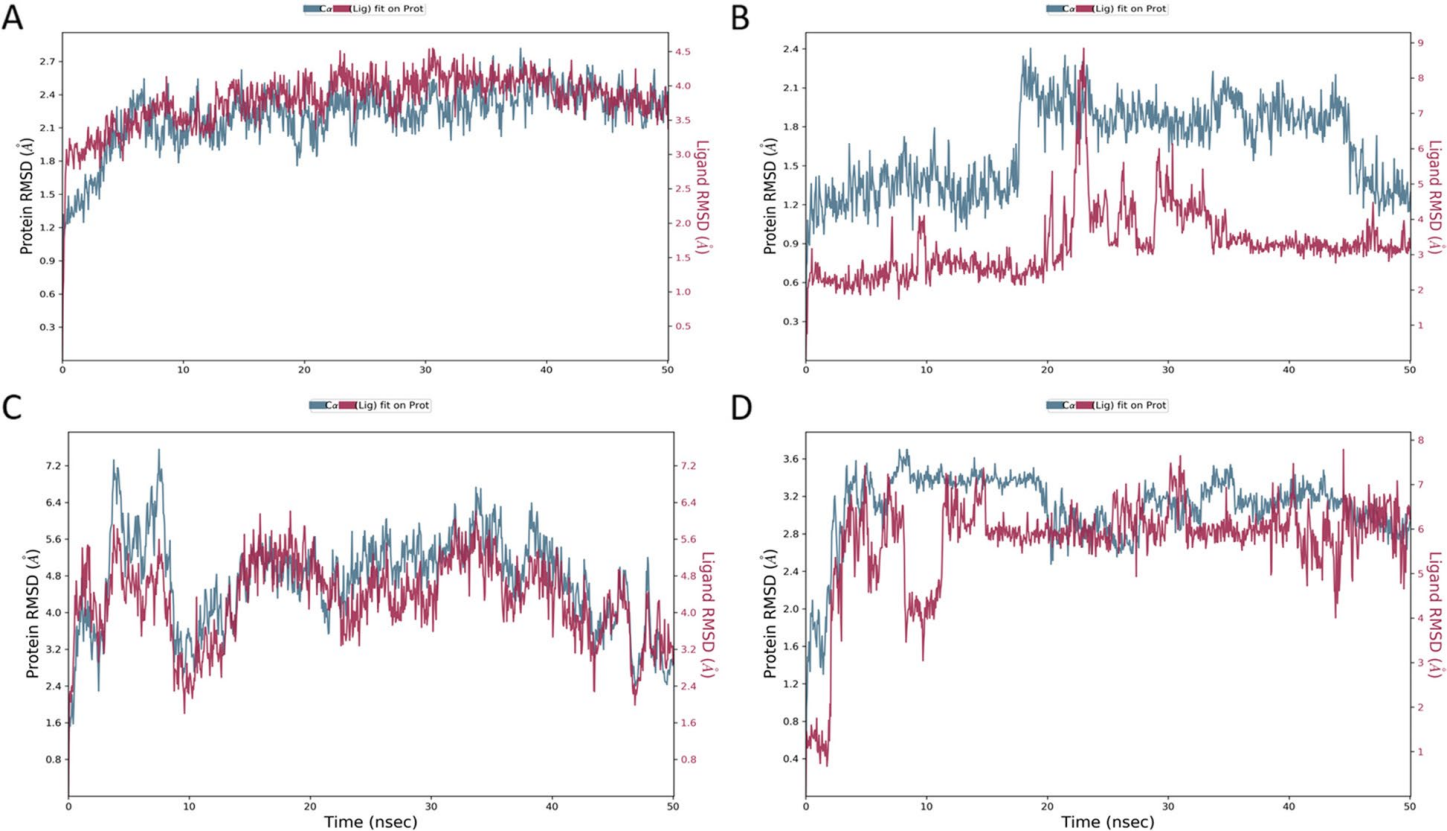
RMSD analysis of MD simulation trajectories generated from the interaction of.beta.d-Mannofuranoside, O-geranyl and backbones of proteins: A. tyrosyl-tRNA synthetase (PDB ID: 1JIJ) B. DNA gyrase (PDB ID: 3TTZ) C. Penicillin-Binding Protein 2X (PBP2X) (PDB ID: 5OJ0), and D. Penicillin-binding protein 4 (PBP4) (PDB ID: 1TVF), during 50 ns simulation time. The ligand is shown in red, and alpha carbon atoms of protein backbones are shown in blue color

**Fig. 5.**
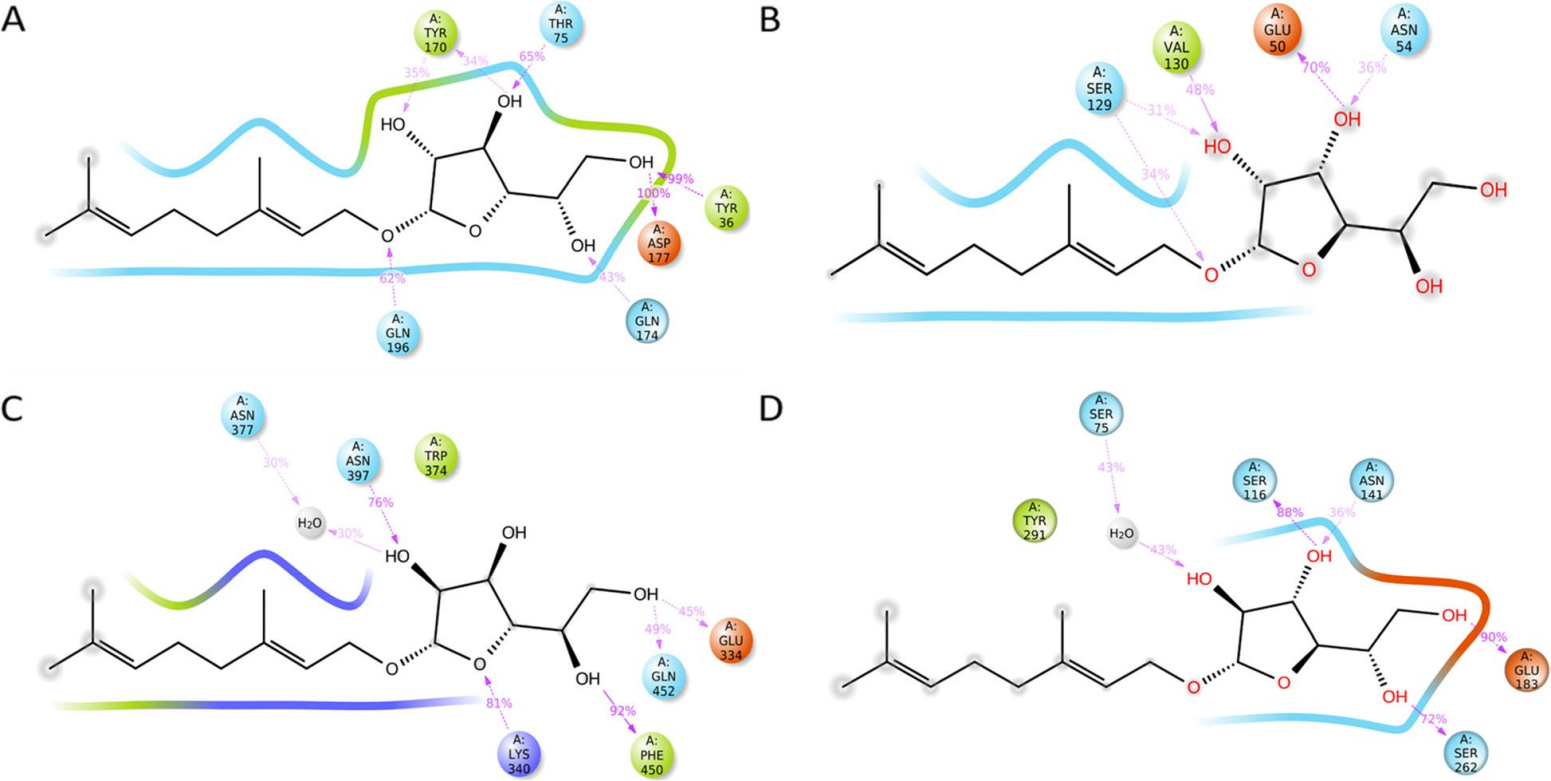
2D interaction of.beta.d-Mannofuranoside, O-geranyl (colored black in the center), and the proteins binding sites residues, showing the percentage of H-bonds (violet) during 50 ns of MD simulation time. Active site residues are colored red (negatively charged), deep blue (positively charged), green (hydrophobic), pale yellow (glycin), pale blue (polar), and water molecules (gray). A. tyrosyl-tRNA synthetase (PDB ID: 1JIJ) B. DNA gyrase (PDB ID: 3TTZ) C. Penicillin-Binding Protein 2X (PBP2X) (PDB ID: 5OJ0), and D. Penicillin-binding protein 4 (PBP4) (PDB ID: 1TVF)

For predicting the interaction stability of ligand and protein complexes, a 50 ns simulation run was performed for the complex of.beta.d-Mannofuranoside, O-geranyl and tyrosyl-tRNA synthetase (PDB ID: 1JIJ), which showed the highest stability during the whole simulation time. The ligand was aligned with protein from the first 5 ns simulation until the end (50 ns) (Fig. 4A). The strongest molecular interaction between ligand and O-geranyl and tyrosyl-tRNA synthetase amino residues was found with Asp117 and Tyr36 (Fig. 5A). Whereas in Fig. 4B, which shows the interaction of.beta.d-Mannofuranoside, O-geranyl and DNA gyrase (PDB ID: 3TTZ), the ligand diverged within less than 3 Å, and with small fluctuation from 25-30 ns simulation time. The Glu50 of DNA gyrase showed 70% stable interaction during 50 ns MD simulation (Fig. 5B). The RMSD plot in Fig. 4C indicates the 50 ns trajectory of the complex of.beta.d-Mannofuranoside, O-geranyl and PBP2X (PDB ID: 5OJ0) revealed the high stability of the complex in which the ligand and protein aligned throughout the whole simulation time. The Phe450, Lys340, and Asn397 of the PBP2X had the most stable interaction during the simulation time (Fig. 5C). The complex of PBP4 (PDB ID: 1TVF) and.beta.d-Mannofuranoside, O-geranyl was stable within 1.2 Å, from 2.4–3.6 Å, this stability occurred after 15 ns simulation time (Fig. 4D). Three residues (Glu83, Ser116, and Ser262) of PBP4 protein showed more than 70% stable interaction with the ligand during the simulation period (Fig. 5D).

### Drug likeness, ADME, and Toxicity prediction

In silico tool is used for prediction of.beta.d-Mannofuranoside, O-geranyl drug-likeness, absorption and distribution, Ames test, and carcinogenicity. As shown in Table 4, the compound showed some side effects with remarkable potential to be developed as an antibacterial agent.

**Table 4:** Drug likeness, ADME, and Toxicity prediction of.beta.d-Mannofuranoside, O-geranyl

Drug likeness Prediction	
Rule of five	Qualified
Lead like rule violation	0
Rule of five violation	0
CMC like rule	Qualified
CMC like rule violation	0
MDDR like rule	At the mid structure
MDDR like rule violation fields	No rings
MDDR like rule violation	2
WDI like rule	Out of 90% cutoff
WDI like rule violation	2
WDI like rule violation fields	Balaban_index_JX, kier_alpha_03
**ADME Prediction (Absorption and distribution)**	
HIA	71.06%
Caco-2	1.6
MDCK	83.2
Skin permeability	-3.9
Blood–brain barrier penetration (BBB)	0.16
Plasma_Protein_Binding (%)	72
**Toxicity Prediction (**Ames test and carcinogenicity)	
Ames_test	Mutagen
TA100_10RLI	Negative
TA100_NA	Positive
TA1535_10RLI	Positive
TA1535_NA	Negative
Carcinogenicity (Mouse)	Positive
Carcinogenicity (Rat)	Negative

### Minimum Inhibitory Concentration (MIC)

The MIC revealed the.beta.d-Mannofuranoside, O-geranyl compound had good activity (MIC 32 μg/ml and 64 μg/ml) against *S. aureus* (ATCC 25,923) and *E. coli* (ATCC 25,922) respectively (Supplementary figure S4).

## Discussion

Plants are now one of the most important sources for finding a new bioactive molecule to treat human diseases caused by pathogenic bacteria [1, 7–9] since more than 80% of people worldwide depend on herbal medicine for their basic healthcare requirements [29]. Neem trees have received worldwide attention as holy trees with remarkable therapeutic benefits such as immunomodulatory, anticancer, antibacterial, and hepatoprotective properties [30]. Neem activity could be due to the presence of a wide array of bioactive phytoconstituents, which include fatty acids, flavonoids, carbohydrates, anthocyanin, cardiac glycosides, phenols, and alkaloids [31]. The present analysis indicates the presence of different phytochemicals in neem extract, including fatty acids, pyridine derivatives, coumarins, and hydrocarbons. Fatty acids have a variety of health benefits and are commonly employed in the pharma industry, and they also have strong antioxidant properties [32]. The coumarin (benzoofuran, 2, 3-dihydro-) was reported to be anti-inflammatory [33].

Neem extract is rich in antimicrobial phytoconstituents such as alkaloids, glycosides flavonoids, phenolic compounds, steroids, triterpenoids, carotenoids, and tetra-triterpenoids azadirachtin [34]. In this study, the GC–MS analysis revealed the presence of 30 phytochemicals in the methanolic extract of neem leaves, the top detected phytochemical compounds were reported previously with antibacterial activity: 1,5-Anhydro-2-deoxy-L-arabino-hex-1-enitol [35], 3,7,11,15-Tetramethyl-2-hexadecane-1-ol [36] 1,3-Propanediol,2-(hydroxymethyl)-2-nitro- [37], n-Hexadecanoic acid [38],.beta. d-Mannofuranoside, O-geranyl [27], and phytol [39].

The methanolic extract of neem leaves used in this study had shown a potent antibacterial activity on different bacteria types. The antibacterial activity of crude neem extract was reported previously in many studies worldwide [40–42]. Methanol can extract a broad polarity range of compounds with antimicrobial activity [43, 43]. In this study, the activity of neem extract was observed on different uropathogens including *S. aureus, E. coli, K. pneumoniae, Citrobacter* spp.*, E. faecalis, P. aeruginosa, Proteus* spp.*,* and *S. epidermidis.* The activity of neem leaves ethanolic extract on urine isolates was documented in Sudan [18, 45], Pakistan [46], and India [47]. Additionally, Okemo et al*.* [48] and Pokhrel et al. [40] reported that the crude extract of the neem plant was very effective against *S. aureus* and *E. coli*. Our results are different from those obtained by Francine and his colleagues [49] in Rwanda. They reported the activity of neem methanolic extract only on *S. aureus* but not on *E. coli.* This variation could be due to differences in neem plants' active constituents and due to differences in environment, genetic factors, and climates [50, 51].

The lowest concentration of neem extract reported with bacterial activity was 3.125%, which showed zones of inhibition of more than 10 mm on *P. aeruginosa, K. pneumoniae, Citrobacter* spp., and *E. coli,* and 8 mm on *Proteus* spp. and *Staphylococcus* spp. These findings are better than Faujdar et al. [47], who also used the same concentrations of neem methanolic extract as in our study, and reported at 6.25 mg/dl concentration a 7 mm zone of inhibition on *E. coli* and *Proteus* spp, and 0 mm on *P. aeruginosa*. Our finding is in concordance with a previous study conducted in Sudan, in which the 6.25 mg/dl concentration was more active on *P. aeruginosa,* and *K. pneumoniae* [45]. This could be due to the presence of the same phytoconstituents in neem plants grown in our environment [18, 50, 51].

Our study showed a high activity of neem extract on bacteria resistant to B-lactam, quinolones, and aminoglycosides, which is consistent with previous findings [45, 47]. Although neem was active on pathogenic *E. coli* and *S. aureus*, we noticed better activity on the control strains of *E. coli* and *S. aureus* than on the pathogenic ones. This could be due to the presence of resistance mechanisms in pathogenic bacteria such as efflux pumps [52, 53].

The molecular docking study showed that.beta.d-Mannofuranoside, O-geranyl had potent activity on essential bacterial proteins. The.beta.d-Mannofuranoside, O-geranyl showed hydrogen bonds with residues that are closely interacted with active sites [54]. The Tyr36, Asp40, Tyr170, and Asp177 of *S. aureus* tyrosyl-tRNA synthetase protein was documented to also have hydrogen bonds with the known co-crystallized protein inhibitor (SB-239629). This activity is concise with our in vitro study and with another in vitro study, in which the authors identified mangrove associates extract with good antibacterial activity, the GC–MS analysis of this extract revealed the presence of a high concentration of.beta.d-Mannofuranoside, O-geranyl [27].

We further evaluated the docking complexes' stability using molecular dynamic simulation, which showed the stability of these complexes during simulation time (50 ns). The ligand was aligned with protein backbones, with a fluctuation of less than 3 Å at most of the simulation period. Usually, changes of the order of 1–3 Å are perfectly acceptable [55]. During the simulation, time residues (Lys340, Trp374, Asn397, and Gln452) of penicillin-binding protein 2X (PDB ID: 5OJ0) showed interaction with protein inhibitor (cefepime) showed a stable interaction with.beta. d-Mannofuranoside, O-geranyl, indicates stability is maintained [56]. On the other hand, the active compounds of neem (.beta.d-Mannofuranoside, O-geranyl) showed a stable interaction with *S. aureus* penicillin-binding protein 4 (PDB ID: 1TVF); the compound formed a stable water bridge with the catalytic residue of the SXXK motif of the penicillin-binding protein 4 [57].

## Conclusion

Methanolic extract of *A. indica* leaves revealed a potent antimicrobial activity to different types of gram-negative, gram-positive, and control strains. In vitro and in silico experiments revealed the.beta. d-Mannofuranoside, O-geranyl is the most active compound on control strains and different bacterial essential proteins. Using in silico ADME/T prediction, the compound passed 5 rules of drug-likeness properties, so it could be further processed for animal testing and clinical trials for its possible use as an antibacterial agent with commercial values. Moreover, the GC–MS analysis of neem extract revealed the presence of a large number of bioactive components, and the most common were fatty acids (11), hydrocarbons (9), pyridine derivatives (2), and aldehydes (2), which played different biological activities in addition to their nutritional benefits. Based on these findings, the neem plant could help us produce safe and effective medications for a variety of diseases. More in-depth research into these identified phytochemicals will aid pharmaceutical explorations.

## Materials and methods

### Collection and identification of bacterial isolates

A total of 130 urine samples indicated for urine culture and sensitivity testing were collected randomly from patients at East Nile Hospital and Ribat University Hospital in Khartoum state, from January to April 2017. The urine samples were cultured on Cystine Lactose Electrolyte Deficient (CLED) agar media (Hi-Media laboratories PV + Ltd, India), and the clinical isolates were identified using conventional biochemical tests [58].

### Antibacterial susceptibility testing

The Kirby-Bauer disk diffusion method [59] was used to test isolated organisms against various antibiotics including ceftazidime (30 mcg), imipenem (10 mcg), gentamicin (10 mcg), cotrimoxazole (25 mcg), and ciprofloxacin (10 mcg) (Hi-Media labs PV + Ltd, India) (5mcg). Results were interpreted according to the Clinical and Laboratory Standard Institute (CLSI) document M100 [59]. The following strains were used for quality control: *S. aureus* (ATCC 25,923) and *E. coli* (ATCC 25,922), to assess the media and antimicrobials disk efficiency.

### Plant collection and extraction

Fresh leaves of wild neem were collected from Algazira (Alkamleen city) in central Sudan in March 2017. Leaves were collected from the same tree into clean, dry, labeled plastic bags. Samples were kept frozen at -80 °C until the time of their use [60]. A taxonomist authenticated the plant at Medicinal and Aromatic Plants and Traditional Medicine Research Institute (MAPRI) National Center for Research, Khartoum, Sudan. At MAPRI herbarium, a voucher sample (No. MAP/2017/4) was deposited. The collected leaves were washed and rinsed to remove dust and other impurities. They were then air-dried and then a total of 50 g of leaves were grounded using a mortar and pestle (Supplementary figure S1), 80% methanol was then used to soak the leaves for three days with daily filtration and evaporation. Then by using a rotary evaporator apparatus under reduced pressure, the solvent was evaporated to dryness [61].

### Antibacterial activity of neem extract

The antibacterial activity of neem leaves was tested using the agar well diffusion method on Muller Hinton Agar (MHA) medium against the isolated bacteria and control strains (*S. aureus* (ATCC 25,923) and *E. coli* (ATCC 25,922)). Three colonies with similar features were dissolved in 1 mL normal saline and turbidity adjusted to 0.5 McFarland. The isolates were then streaked on the surface of the MHA plate with a sterile swab. Using a cork borer, 6 mm wells were created aseptically on MHA. At sterile conditions, 100 µl of each 50, 25, 12.5, 6.25, 3.125, and 1.5% concentrations of neem extract were poured into media wells [62]. The plates were placed refrigerated for 1 h to allow for extract diffusion before being incubated at 37 °C for 24 h. Methanol alone was used as a negative control. The zone of inhibition was measured (in mm), and the mean was calculated [63]. Three replicates were carried out for the activity of extracted neem against tested organisms. Then the data were presented as mean and standard deviation.

### Phytochemical screening of *A. indica* (neem) extract

The GM-MS method was used to conduct a qualitative and quantitative characterization of neem extract, using the model (GC–MS-QP2010-Ultra) from Shimadzu Company, Japan, with a capillary column Rtx®-5MS column (30 m, 0.25 mm, 0.25 µm) [64]. The split mode was used for sample injection, and operated in electron ionization (EI) mode at 70 eV, inflow rate of 1.69 ml/min. Helium gas was used as carrier gas. The injector temperature was set at 300 °C, the temperature of the ion source was 200 °C, and 250 °C was used as interface temperature. The oven temperature program was as follows: the initial temperature at 50 °C rising at 7 °C /min to 180 °C, then the rate changed 10 °C/min reaching the final temperature at 280 °C with 2 min as hold time. In a total 22 min run, the sample was analyzed by the scan mode in a range of 40 to 500 m/z charges to ratio. The neem extract's components were identified by comparing the retention times and mass fragmentation patents with the National Institute of Standards and Technology (NIST) library, and then the results were recorded [65, 66].

## In silico analysis

### Molecular docking

#### Proteins selection and preparation

The crystal structures of four essential bacterial proteins were obtained from RCSB PDB database [67] according to their essential role in bacterial cell wall synthesis and protein production in most of our studied isolates, and according to published data [68–71]. These proteins were *Staphylococcus aureus* tyrosyl-tRNA synthetase (PDB ID: 1JIJ), DNA gyrase (PDB ID: 3TTZ), Penicillin-Binding Protein 2X (PBP2X) from *Streptococcus pneumoniae* (PDB ID: 5OJ0), and penicillin-binding protein 4 (PBP4) from *Staphylococcus aureus* (PDB ID: 1TVF). The proteins’ 3D structures were prepared with the Protein Preparation Wizard in Maestro using the default setting. For validation of the docking method, the co-crystalized ligands, SB-239629, 07 N, and cefepime, with their respective structures (1JIJ, 3TTZ, and 5OJ0, respectively), were redocked again using Maestro software [72].

### Ligands preparation

The structures of compounds identified by GC–MS (Table 3) were obtained from NCBI PubChem and ChemSpider databases. The ligand*'*s energy was minimized using LigPrep (Schrodinger software, version 2020–3).

### Molecular docking

Proteins’ active sites were predicted using the Receptor Grid Generation module in Schrodinger. The grids were specified around the co-crystalized ligands or using the SiteMap module to predict *S. aureus* (PDB ID: 1TVF) protein according to published data [69]. The prepared molecules were docked on protein active sites using extra precision (XP) docking of Schrödinger Maestro software [72]. Ligands were set flexible while proteins were set rigid.

### Molecular Dynamic (MD) simulation

Desmond package in the Schrödinger Maestro software [72] was used for MD simulation. The complexes with the best interaction and docking energy were first solvated into the TIP3P water model, an orthorhombic box with boundary 10 Å beyond any of the complex's atoms. Charges were neutralized and OPLS3e force field was used. The particle mesh Ewald method was used for the calculation of long-range electrostatic interactions [73] and cutoff of 12 Å. The molecular dynamic simulation was done in the NPT ensemble at a temperature of 300 K and 1.013 bar pressure over 50 ns and 30 ps for trajectory and 100 ps relaxation time. The trajectories were recorded in 50 ps intervals. After job completion, Root Mean Square Deviation (RMSD) and Root Mean Square Fluctuation (RMSF) were used to examine complexes' stability.

### Drug-likeness, ADME & Toxicity Prediction Studies

The ADME profiles and toxicity analysis were carried out using PreADMET (http://preadmet.bmdrc.org).

### Minimum Inhibitory Concentration (MIC)

Antibacterial activity of the most stable ligand (.beta.d-Mannofuranoside, O-geranyl) from the in silico study was evaluated using in vitro method. The compound was purchased from Apollo Scientific (UK), the compound ID: MolPort-019–937-357, purity 95%, and the Molecular weight was 316.394. The MIC of the compound was evaluated against *S. aureus* (ATCC 25,923) and *E. coli* (ATCC 25,922) using the microtitre broth dilution method [74]. A twofold serial dilution of the compound was prepared in broth media (Muller-Hinton), using 96-well microplates flat-bottom plates. One 100 *μ*L of culture media containing bacterial growth adjusted to 5–10^5^ CFU/ml was poured into each well. The MIC of the compound was determined at a concentration ranging from 0.5 to 256 μg/ml [75].

## Supplementary Information

**Additional File1.** 

## Data Availability

All data are included in the manuscript, any additional information needed contact the corresponding author.

## Abbreviations

ATCC: American Type Culture Collection
WHO: World Health Organization
GC-MS: Gas chromatography-mass spectrometry
CID: Compound ID
HIA: Human intestinal absorption
CMC: Comprehensive medicinal chemistry
MDDR: MDL Drug Data Report
WDI: World Drug Index
MDCK: Madin-Darby Canine Kidney Cell Permeability
RMSD: Root Mean Square Deviation
RCSB PDB: Structural Bioinformatics Protein Data Bank
ADME/T: Absorption, Distribution, Metabolism, Excretion and Toxicity
