# Supplementary material for: In-vitro and in-silico antibacterial activity of Azadirachta indica (Neem), methanolic extract, and identification of Beta.d-Mannofuranoside as a promising antibacterial agent
Source: BMC Plant Biol. 2022 May 25;22:262. doi: 10.1186/s12870-022-03650-5 (PMC9131563; doi:10.1186/s12870-022-03650-5)
Supplement: Supplementary file 1 — Additional File1. [file 12870_2022_3650_MOESM1_ESM.docx]

Figure S1. Collection of *A. indica* and drying in shadow (left) and ground by using a mortar and pestle (right).


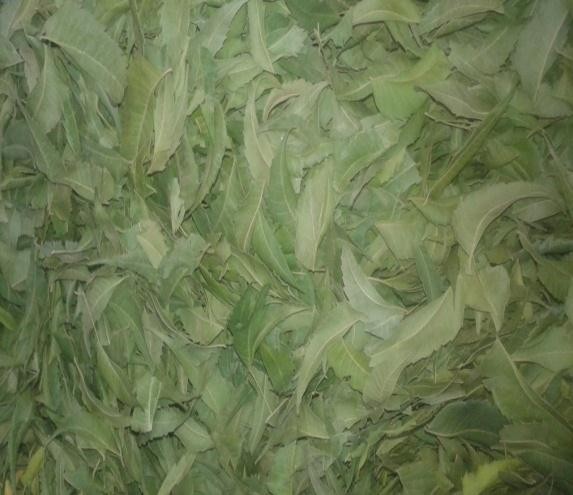

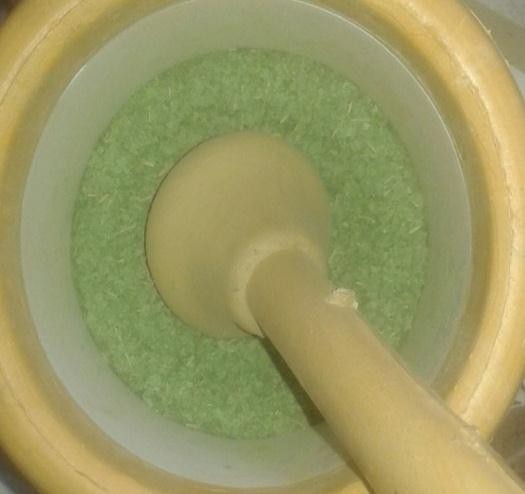


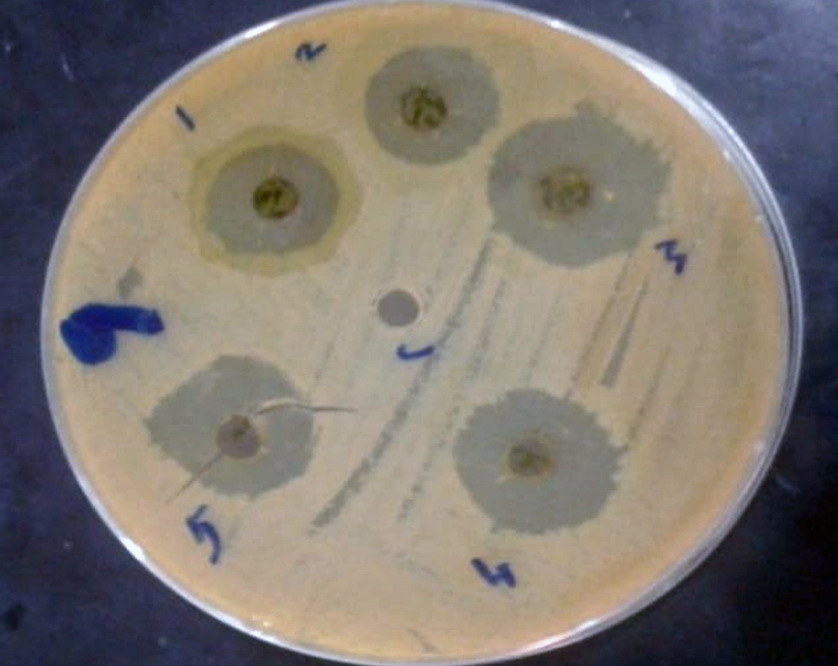


Figure S2. The activity of A. indica leaves methanolic extract on a pathogenic E. coli at different concentrations, the numbers 1, 2, 3, 4, and 5 corresponding to concentrations 50, 25, 12.5, 6.25, and 3.125, % respectively, C (at center) indicates for negative control.


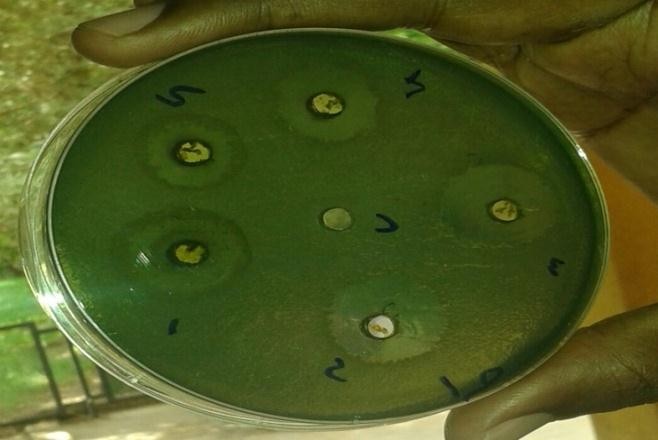


Figure S3. The activity of *A. indica* leaves methanolic extract at different concentrations, the numbers 1, 2, 3, 4, and 5 corresponding to concentrations 50, 25, 12.5, 6.25, and 3.125% respectively, C indicates negative control. Antibacterial activity of neem extract on *P. aeruginosa* isolate.


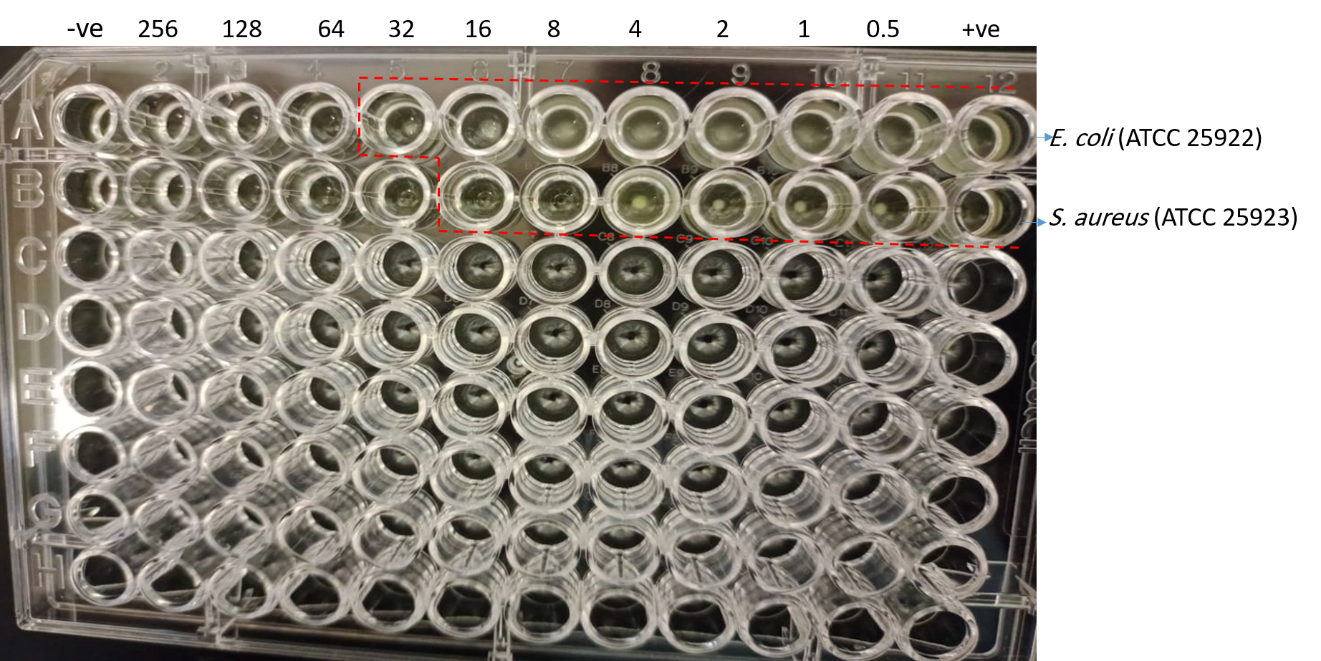


Figure S4. Determination of the minimum inhibitory concentration (MIC) of beta.d-Mannofuranoside, O-geranyl compound using micro-plates. Ten concentrations series were used in 2 times dilution fold. The red dashed lines show the borders of the last wells showing growth. The first wells on the left were used for negative (-ve) control (compound without bacterial growth), and the right wells were used for positive control (bacterial growth without compound).
